# Core self-evaluation and innovative behavior: mediating effect of error orientation and self-efficacy of nurses

**DOI:** 10.3389/fpsyg.2023.1298986

**Published:** 2023-12-05

**Authors:** Guiyue Ma, Zhihao Han, Xiaoqin Ma

**Affiliations:** ^1^School of Nursing, Zhejiang Chinese Medical University, Hangzhou, China; ^2^School of Nursing, Anhui University of Chinese Medicine, Hefei, China

**Keywords:** nurse, core self-evaluation, error orientation, self-efficacy, innovative behavior, mediating effect

## Abstract

**Background:**

Innovation plays a crucial role in advancing nursing and healthcare. Despite its significance, there is a paucity of research examining the interplay among nursing innovative behavior, core self-evaluation, error orientation, and self-efficacy. This study, grounded in Bandura’s social cognitive theory, seeks to not only investigate the influence of core self-evaluation on nurses’ innovative behavior but also to elucidate the mediating roles of error orientation and self-efficacy within this relationship. By addressing these dynamics, the research aims to provide a comprehensive understanding of the factors shaping nurses’ innovative behaviors and contribute to the broader discourse on enhancing healthcare practices.

**Design:**

A cross-sectional study using an online questionnaire.

**Setting:**

Participants were recruited from 23 hospitals in 6 provinces and 1 municipality directly under the central government in China, namely Zhejiang, Anhui, Jiangxi, Guangdong, Hebei, Henan, and Shanghai.

**Participants:**

A total of 741 nurses enrolled in the study.

**Methods:**

The participants completed the nurse innovative behavior scale, the core self-evaluation scale, the error orientation questionnaire, and the self-efficacy scale online in 2023. SPSS and AMOS were used for data analysis. The reporting followed the STROBE checklist.

**Results:**

A total of 706 valid questionnaires were collected. A positive core self-evaluation was associated with more innovative behavior, and this relation was partially mediated by error orientation and self-efficacy to avoid failure. Core self-evaluation, error orientation and self-efficacy of nurses had a positive predictive effect on innovation behavior, with the path coefficients at 0.09, 0.23, and 0.39, respectively.

**Conclusion:**

Our study complements the evidence on the mechanism of action between the core self-evaluation and innovative behavior. Our findings have important clinical implications for promoting innovative behavior in nurses.

## 1 Introduction

Nurses play a critical role in delivering high-quality healthcare services and ensuring positive patient outcomes. In recent years, nursing innovation research has played a pivotal role in driving progress and excellence in healthcare (Pennington and Driscoll, 2019). As frontline caregivers, nurses are uniquely positioned to identify the inefficiencies, gaps, and challenges within the healthcare system. Embracing innovation empowers nurses to explore novel solutions, implement evidence-based practices, and improve patient outcomes (Drury et al., 2023). By encouraging creativity and promoting a culture of continuous improvement, nursing innovation research enhances the quality, safety, and efficiency of healthcare services. Innovative behavior encompasses a dynamic process involving the creation, validation, and implementation of imaginative ideas. These ideas find their application in various domains such as research, management, education, and clinical practices (Asurakkody and Shin, 2018). In this research, measuring innovative behavior involves activities including idea generation, support obtaining and idea realization (Ling et al., 2012). The overall objective of this study is to investigate, through the framework of Bandura’s social cognitive theory, the relationships among nurses’ innovative behavior, core self-evaluation, error orientation, and self-efficacy. By elucidating the interplay between these factors, the study aims to provide essential insights for the nursing and healthcare field, thereby advancing both the theoretical understanding and practical development of innovative behaviors.

### 1.1 Background

#### 1.1.1 Core self-evaluation and innovative behavior

Core self-evaluation, often abbreviated as CSE, finds its origins in the realm of self-perception and psychological well-being. Core self-evaluation, encompassing self-esteem, locus of control, emotional stability, and self-efficacy, forms a fundamental aspect of an individual’s self-perception and overall psychological well-being (Farèiæ et al., 2020). In essence, CSE examines the fundamental beliefs that individuals hold about their own competence and value. It shares strong connections with more commonly recognized self-factors, such as personality traits and characteristics. It helps us understand how individuals view themselves and how these perceptions influence their attitudes, behaviors, and responses, making it a key factor in studies of innovative behavior and overall well-being (Cross et al., 2021). In the context of nursing, the interplay between core self-evaluation and nurse innovation behavior has garnered increasing attention due to its potential implications for enhancing patient care and healthcare outcomes. Core self-evaluation significantly impacts nurses’ attitudes, beliefs, and behaviors (Sulaiman et al., 2021). Research has revealed that nurses with higher core self-evaluation tend to exhibit more positive attitudes toward their own capabilities and self-worth. This heightened sense of self-esteem and self-efficacy often leads to greater confidence in their problem-solving abilities and a willingness to embrace challenges in their practice. Consequently, nurses with robust core self-evaluation are more inclined to generate creative ideas, seek support, and implement innovative practices in various aspects of nursing, such as research, management, education, and clinical care (Chen et al., 2022). Considering the importance of core self-evaluation in shaping nurses’ propensity for innovation, it is hypothesized:

Hypothesis 1: Nurses with higher core self-evaluation are positively associated with innovative behavior.

This hypothesis underscores our exploration of the link between nurses’ core self-evaluation and their inclination toward innovative practices, contributing to the broader understanding of factors influencing nursing innovation and, ultimately, patient care quality and healthcare outcomes.

#### 1.1.2 Error orientation and innovative behavior

In terms of nurses’ innovative behavior, error orientation and self-efficacy are key factors (Farnese et al., 2022). Error orientation refers to one’s attitude toward handling errors, encompassing acceptance, learning, and enhancing self-efficacy through errors. Positive error orientation portrays errors as self-learning opportunities, improving self-efficacy and fostering innovative behavior. Such orientation prompts nurses to respond to errors through self-reflection, boosting core self-evaluation and encouraging innovative behavior (Yun and Du, 2019). Studies indicate that nurses who view errors as opportunities for learning and growth are more likely to actively participate in innovation. This positive perspective on errors fosters a mindset that drives innovative solutions and practice improvement. Viewing errors as valuable learning experiences empowers nurses to explore new ideas, methods, and creative solutions. Reframing errors as chances for improvement motivates nurses to seek innovative approaches for better patient care and outcomes (Xu et al., 2022). On the other hand, nurses with negative error orientation might avoid risks and new methods due to fear, limiting their innovative engagement and contributions (Zhou et al., 2023). Considering the pivotal role of error orientation in shaping nurses’ inclination toward innovation, we propose:

Hypothesis 2: Nurses with a positive error orientation are positively associated with innovative behavior.

This hypothesis aims to examine the correlation between nurses’ positive error orientation and their engagement in innovative practices, contributing to our understanding of the factors influencing nursing innovation and its impact on patient care and outcomes.

#### 1.1.3 Self-efficacy and innovative behavior

Self-efficacy, a concept proposed by psychologist Bandura (Bandura et al., 1999), refers to an individual’s belief in their ability to successfully perform specific behaviors. Within the nursing context, self-efficacy plays a crucial role in shaping nurses’ attitudes, behaviors, and performance, including their inclination toward innovative practices. Numerous studies have highlighted the positive impact of self-efficacy on nurse innovation behavior. For instance, studies by Ng and Lucianetti (2016) found that nurses with higher levels of self-efficacy tend to engage in more innovative practices and demonstrate a greater willingness to generate creative ideas and seek solutions to complex challenges in their daily work. Self-efficacy plays a fundamental role in nurturing nurse innovation behavior, empowering nurses to approach their work with confidence and a proactive attitude toward finding innovative solutions. Recognizing the pivotal influence of self-efficacy on nurses’ propensity for innovation, we posit:

Hypothesis 3: Nurses with higher self-efficacy are positively associated with innovative behavior.

This hypothesis aims to explore the relationship between nurses’ elevated levels of self-efficacy and their involvement in innovative practices, contributing valuable insights to our understanding of the factors influencing nursing innovation and its broader impact on patient care and the nursing profession.

#### 1.1.4 Core self-evaluation, error orientation, self-efficacy, and innovative behavior

Understanding the intricate interplay among core self-evaluation, error orientation, self-efficacy, and nurses’ innovative behavior is essential for comprehending the multifaceted nature of nursing practices. While core self-evaluation, error orientation, and self-efficacy are individually recognized as influential factors in fostering nurse innovation (Ng and Lucianetti, 2016; Farnese et al., 2022), the theoretical foundation supporting the mediation hypothesis requires further elucidation. Previous research has demonstrated the interconnectedness of these constructs within the broader framework of Bandura’s social cognitive theory. Core self-evaluation serves as the lens through which individuals perceive their own competence and worth, influencing their attitudes and behaviors. This perception, in turn, can impact error orientation and self-efficacy. Positive core self-evaluation may lead to a more constructive error orientation, where mistakes are viewed as opportunities for learning and growth. Simultaneously, it can enhance self-efficacy, fostering a belief in one’s ability to navigate challenges effectively. Studies by Yun and Du (2019) and Xu et al. (2022) suggest that the relationship between core self-evaluation, error orientation, self-efficacy, and innovative behavior is dynamic. Positive core self-evaluation may enhance both error orientation and self-efficacy, contributing to a nurse’s willingness to engage in innovative practices. Conversely, negative core self-evaluation may hinder the development of a positive error orientation and impede the belief in one’s ability to innovate. Given this theoretical backdrop, we propose:

Hypothesis 4: Error orientation and self-efficacy play a mediating role between core self-evaluation and nurses’ innovative behavior.

This hypothesis aims to explore the intricate pathways through which core self-evaluation influences nurse innovation, considering the mediating roles of error orientation and self-efficacy. By examining these relationships, we seek to contribute nuanced insights into the underlying mechanisms shaping nursing innovation and its broader impact on patient care and the nursing profession.

## 2 Materials and methods

### 2.1 Design and sample, and settings

This study used convenience sampling with a cross-sectional study. The participants were recruited from 23 hospitals in 6 provinces and 1 municipality directly under the central government in China, namely Zhejiang, Anhui, Jiangxi, Guangdong, Hebei, Henan, and Shanghai. The current researchers randomly selected two departments in each hospital to investigate all the nurses working in these sections. The inclusion criteria were (1) with a Nurse Professional Qualification Certificate; (2) with ≥6 months’ practice time. The exclusion criteria are as follows: (1) internship or probationary nurses; and (2) nurses for sick or maternity leave. A total of 741 registered nurses were invited to participate in this survey, and 706 valid questionnaires were received, resulting in a participation rate of 95.28%.

### 2.2 Measures

The research tools used in this study include five components: (a) demographic information, (b) core self-evaluation scale, (c) error orientation scale, (d) self-efficacy scale, and (e) innovative behavior questionnaire.

#### 2.2.1 Demographic information of the characteristics

The demographic information sheet was designed by the researchers. Previous studies were used to determined potential demographic variables that may affect innovative behavior. Eventually, 13 variables were considered, namely, gender, age, marital status, education level, monthly per capita household income, employment method, technical title, position, total working years (year), whether to publish/patent/apply as the first author, the level of the hospital, types of hospitals including those that focus on Western medicine, traditional Chinese medicine, or a combination of both, as well as the nature of the hospital, which can be either a general hospital or a specialty hospital.

#### 2.2.2 Core self-evaluation scale

Core self-evaluation scale was adapted from Judge and Bono (2001). This scale is a single-dimensional self-assessment scale consisting of 10 items, each rated on a 5-point Likert scale. The response options range from 1, indicating “completely disagree,” to 5, indicating “completely agree,” with 2 representing “disagree,” 3 as “neutral,” and 4 as “agree.” It is essential to recognize that higher scores on this scale correspond to a more pronounced sense of core self-evaluation. A high score indicates that an individual has a strong belief in their self-worth, confidence in their abilities, and a positive perception of themselves. The revised core self-evaluation scale demonstrated robust reliability, with a Cronbach’s alpha coefficient of 0.83. This coefficient measures the internal consistency of the scale, indicating how closely the items in the scale are related to each other in assessing the same construct. Furthermore, the split-half reliability of the scale was determined to be 0.84, indicating the extent to which the scale produces consistent results when divided into two equal halves. In this study, the Cronbach’s alpha for the total scale was calculated at 0.86, affirming the scale’s reliability for the research purposes.

#### 2.2.3 Error orientation questionnaire

The error orientation questionnaire, developed by Rybowiak et al. (1999), was translated and revised by Weiwei (2008), demonstrating good reliability and validity. The questionnaire comprises eight dimensions: error competence (referring to individuals’ immediate knowledge and ability to handle errors when they occur), learning from errors (reflecting the knowledge gained from errors to improve future work), error risk taking (indicating individuals’ flexibility and openness in dealing with errors), error strain (representing the stress caused by errors), error anticipation (acknowledging that even experts can make mistakes or adopting a cautious approach due to fear of errors), covering up errors (viewing errors as threats and being unwilling to take responsibility), error communication (seeking help from others when encountering errors and sharing one’s errors, solutions, and prevention methods with others), and thinking about errors (analyzing the causes of errors and potential solutions after they occur). Responses are measured on a 5-point Likert scale, with scores ranging from 1 (completely inconsistent) to 5 (completely consistent). For the dimensions of error strain and covering up errors, reverse scoring is applied. The questionnaire consists of 36 items with a Cronbach’s alpha coefficient of 0.88. In this particular study, the total scale’s Cronbach’s alpha value is 0.80.

#### 2.2.4 Self-efficacy scale

The self-efficacy scale utilized in this study was originally developed by Schwarzer (1997) and subsequently translated and adapted for use by Chinese scholar (Zhang, 2005). The scale is a unidimensional instrument, consisting of 10 items. Participants rate their responses on a 4-point Likert scale, ranging from 1 (completely wrong) to 4 (absolutely right). The total self-efficacy score is derived from combining the item scores, with higher scores indicating higher levels of self-efficacy among the nurses. The self-efficacy scores are categorized as follows: scores between 1 and 1.9 indicate low self-efficacy levels, scores between 2 and 2.9 indicate moderate self-efficacy levels, and scores between 3 and 4 indicate high self-efficacy levels. The scale demonstrates good internal consistency, with a Cronbach’s alpha reliability coefficient of 0.87 for the Chinese version. Additionally, the test-retest reliability is 0.83, indicating the scale’s stability over time. In this particular study, the total scale’s Cronbach’s alpha value is 0.93.

#### 2.2.5 Nurse innovative behavior scale

The innovative behavior questionnaire, developed by Ling et al. (2012), assesses three dimensions: idea generation, support obtaining, and idea realization. It consists of ten items, such as “I had the willingness to solve problems.” Participants respond on a 5-point Likert scale, ranging from 1 (completely inconsistent) to 5 (completely consistent), resulting in a total score between ten and 50 points. A higher total score indicates a higher level of active innovative behavior. The Cronbach’s alpha for the original questionnaire was 0.88, while in this study, it yielded a Cronbach’s alpha of 0.94.

### 2.3 Ethical considerations

This study had gained approval from the Ethics Committee of Zhejiang Chinese Medical University (20230714-3).

### 2.4 Data collection

The data collection took place in July 2023. We distributed the questionnaires in an online survey format. To increase the likelihood of valid responses to the questions, participants were informed that all answers would remain anonymous. This information was included in the informed consent. Participants were informed that the questionnaire typically required approximately 10–15 min for completion. They were also reminded that their participation was entirely voluntary, and they had the right to decline participation or discontinue the survey at any point. The electronic questionnaire was designed to allow participants to skip questions they did not wish to answer without any consequences. A total of 741 questionnaires were distributed. Out of the total participants, 35 individuals (4.72%) were excluded from the initial dataset. This exclusion was necessary because it was observed that their responses to different questionnaire items exhibited a noticeable pattern or similarity. In the end, 706 sets of questionnaires were collected and analyzed. To address concerns about potential duplicate responses, we employed several strategies to ensure the integrity of the data. We implemented technical measures in the electronic questionnaire to prevent multiple submissions from the same participant. Additionally, participants were reminded that the survey was designed for single-use only, and the anonymous nature of the survey minimized the likelihood of participants attempting to complete it more than once.

### 2.5 Statistical analysis

The data analysis involved both descriptive and inferential statistics using Statistical Package for Social Sciences (SPSS, version 27.0). Descriptive statistics were utilized to summarize the sample characteristics and average scores for core self-evaluation, error orientation, self-efficacy, and innovative behavior. An independent sample *T*-test was conducted to compare the main variables, and multiple linear regression was used to analyze the innovative behavior of nurses. Pearson correlations were used to test the relationships among core self-evaluation, error orientation, self-efficacy, and innovative behavior. Structural equation modeling (SEM) was used to analyze the hypothetical models. SEM is a comprehensive method for testing relationships among variables. The analysis of Moment Structures (AMOS, version 28.0) was used as part of the SEM analysis. The reporting followed the STROBE checklist.

## 3 Results

### 3.1 Demographic characteristics

There were 694 female nurses (98.3%) and 12 male nurses (1.7%). Among them, 547 were married (77.5%) and 159 were unmarried (22.5%). The average age was (33.92 ± 7.49) years. The average working experience was (9.35 ± 5.91) years. Out of the total, 146 individuals (20.7%) had a Junior college and below educational background, 524 individuals (74.2%) had a Bachelor’s degree, and 36 individuals (5.1%) had a graduate or higher degree. In terms of nursing titles, there were 107 nurses (15.2%), 268 primary nurses (38.0%), 271 nurses-in-charge (38.4%), and 60 deputy chief nurses or above (8.5%). In terms of employment method, 309 individuals (43.8%) were under the establishment system, whereas 397 individuals (56.2%) were under the contract system. Table 1 presents the demographic characteristics of the participants.

**TABLE 1:** Socio-demographic and one-way analysis of variance for participants (*N* = 706).

Item	*N*	%	The score for innovative behavior	*t*/*F*	*P*
**Gender**					
Male	12	1.7	38.00 ± 7.34	1.616	0.133
Female	694	98.3	34.55 ± 7.03		
**Age**					
20–30	249	35.3	34.78 ± 7.38	5.079	0.002
31–40	346	49.0	33.80 ± 7.06		
41–50	83	11.8	36.84 ± 5.59		
51–60	28	4.0	36.46 ± 5.81		
**Marital status**					
Unmarried	159	22.5	34.58 ± 6.88	−0.050	0.960
Married	547	77.5	34.62 ± 7.09		
**Education level**					
Junior college and below	146	20.7	34.58 ± 6.94	4.599	0.003
Bachelor’s degree	524	74.2	34.38 ± 7.06		
Master’s degree	33	4.7	37.39 ± 6.13		
Doctoral degree	3	0.4	46.00 ± 4.00		
**Monthly per capita household income**					
<3,000	88	12.5	32.78 ± 7.09	3.613	<0.001
3,001–5,000	254	36.0	33.66 ± 6.96		
5,001–7,000	108	15.3	34.38 ± 7.40		
7,001–10,000	99	14.0	36.21 ± 7.11		
>10,000	157	22.2	36.31 ± 6.33		
**Employment method**					
Establishment system	309	43.8	35.68 ± 6.92	3.613	<0.001
Contract system	397	56.2	33.77 ± 7.03		
**Technical title**					
Nurse	107	15.2	34.76 ± 7.34	7.330	<0.001
Primary nurse	268	38.0	33.59 ± 7.11		
Nurse-in-charge	271	38.4	34.76 ± 6.82		
Deputy chief nurse and above	60	8.5	38.20 ± 5.99		
**Position**					
Not have	486	68.8	33.89 ± 7.01	9.627	<0.001
Nursing team leader/teaching teacher	109	15.4	34.71 ± 6.79		
Deputy head nurse/head nurse	86	12.2	38.15 ± 6.23		
Department head nurse and above	25	3.5	36.00 ± 7.81		
**Total working years (years)**					
1–5	140	19.8	35.46 ± 7.40	4.775	<0.001
6–10	198	28.0	33.47 ± 6.47		
11–15	199	28.2	33.82 ± 7.27		
16–20	74	10.5	35.62 ± 7.83		
>20	95	13.5	36.58 ± 5.82		
**Whether to publish/patent/apply as the first author**					
Yes	229	32.4	36.64 ± 6.78	5.462	<0.001
No	477	67.6	33.64 ± 6.96		
**The level of the hospital**					
Three-level	524	74.2	34.61 ± 6.94	0.778	0.507
Two-level	127	18.0	34.29 ± 7.06		
One-level	10	1.4	32.90 ± 6.10		
Other	45	6.4	35.89 ± 8.28		
**The types of the hospital**					
Western hospital	328	46.5	34.78 ± 7.22	2.431	0.089
Traditional Chinese medical hospital	74	10.5	36.01 ± 6.30		
Hospitals of traditional Chinese and western medicine	304	43.1	34.08 ± 6.98		
**The nature of the hospital**					
General hospital	566	80.2	34.45 ± 7.03	−1.211	0.227
Specialty hospital	140	19.8	35.26 ± 7.08		
Core self-evaluation	–	–	–	4.790	<0.001
Error orientation	–	–	–	4.078	<0.001
Error competence	–	–	–	8.176	<0.001
Learning from errors	–	–	–	7.444	<0.001
Error risk taking	–	–	–	5.515	<0.001
Error strain	–	–	–	2.358	<0.001
Error anticipation	–	–	–	4.463	<0.001
Covering up errors	–	–	–	5.416	<0.001
Error communication	–	–	–	8.175	<0.001
Thinking about errors	–	–	–	6.647	<0.001
Self-efficacy	–	–	–	12.381	<0.001

### 3.2 Scores of core self-evaluation, error orientation, self-efficacy, and innovative behavior among nurses

Based on the analysis, the data for core self-evaluation, error orientation, self-efficacy, and innovative behavior among nurses were found to follow a normal distribution. The core self-evaluation, error orientation, error orientation, self-efficacy, and innovative behavior of the participants were in medium-high level. The specific total score range, total score, and average score for each scale are shown in Table 2.

**TABLE 2:** Scores of core self-evaluation, error orientation, self-efficacy, and innovative behavior among nurses (*N* = 706).

Item	Total score range (scores)	Total score ($\bar{\text{X}}$ ± S)	Average score ($\bar{\text{X}}$ ± S)
Core self-evaluation	10–50	36.20 ± 6.28	3.62 ± 0.63
Error orientation	36–180	123.78 ± 12.20	3.44 ± 0.34
Error competence	4–20	15.66 ± 2.68	3.92 ± 0.67
Learning from errors	4–20	15.14 ± 3.25	3.79 ± 0.81
Error risk taking	4–20	12.19 ± 3.71	3.05 ± 0.93
Error strain	5–25	13.15 ± 3.86	2.63 ± 0.77
Error anticipation	5–25	13.92 ± 3.85	2.78 ± 0.77
Covering up errors	5–25	17.93 ± 4.26	3.59 ± 0.85
Error communication	4–20	15.60 ± 2.72	3.90 ± 0.68
Thinking about errors	5–25	20.20 ± 3.46	4.04 ± 0.69
Self-efficacy	10–40	27.57 ± 5.86	2.76 ± 0.59
Innovative behavior	10–50	34.61 ± 7.04	3.46 ± 0.70
Idea generation	3–15	11.03 ± 2.15	3.68 ± 0.72
Support obtaining	4–20	13.33 ± 3.06	3.33 ± 0.77
Idea realization	3–15	10.25 ± 2.55	3.42 ± 0.85

### 3.3 The status of innovative behavior among nurses

According to Table 3, more than 50% of nurses often demonstrate the willingness to generate solutions to problems and analyze the feasibility of problem-solving methods in practical work. Additionally, over 40% of nurses often utilize resources to explore problem-solving methods, seek approval, support, and participation from colleagues or leaders, apply the implementation plans to their work, and revise those plans as necessary and apply them to their work.

**TABLE 3:** The status of innovative behavior among nurses (*N* = 706).

Item	$\bar{\text{X}}$ ± S	Frequency [*n* (%)]				
		Never	Seldom	Sometimes	Often	Always
Willingness to generate solutions to problems	3.69 ± 0.78	6 (0.85)	40 (5.67)	202 (28.61)	380 (53.82)	78 (11.05)
Utilizing resources to explore problem-solving methods	3.63 ± 0.83	11 (1.56)	42 (5.95)	227 (32.15)	343 (48.58)	83 (11.76)
Analyzing the feasibility of problem-solving methods in practical work	3.71 ± 0.74	1 (0.14)	34 (4.82)	215 (30.45)	375 (53.12)	81 (11.47)
Seeking colleagues’ or leaders’ approval, support, and participation	3.66 ± 0.75	2 (0.28)	34 (4.82)	244 (34.56)	347 (49.15)	79 (11.19)
Conducting research on new methods to gather more information	3.43 ± 0.87	5 (0.71)	95 (13.46)	266 (37.68)	269 (38.10)	71 (10.06)
Seeking funding support for new methods	2.93 ± 1.09	71 (10.06)	184 (26.06)	221 (31.30)	184 (26.06)	46 (6.52)
Developing specific implementation plans for new methods	3.30 ± 0.93	15 (2.12)	120 (17.00)	268 (37.96)	241 (34.14)	62 (8.78)
Applying the implementation plans to work	3.44 ± 0.87	7 (0.99)	93 (13.17)	256 (36.26)	285 (40.37)	65 (9.21)
Revising the implementation plans and applying them to work	3.42 ± 0.89	8 (1.13)	103 (14.59)	246 (34.84)	283 (40.08)	66 (9.35)
Regularly evaluating the effectiveness of the new methods	3.40 ± 0.90	12 (1.70)	98 (13.88)	257 (36.40)	274 (38.81)	65 (9.21)

### 3.4 Correlation analysis among core self-evaluation, error orientation, self-efficacy, and innovative behavior among nurses

Pearson’s r test was constructed to test the correlation of core self-evaluation, error orientation, self-efficacy, and innovative behavior of nurses (Table 4). The results indicated positive correlation among all those variables (*P* < 0.01). This robustly supports the validity of Hypotheses 1, 2, and 3. The observed positive correlations offer compelling evidence, affirming the hypothesized relationships between core self-evaluation, error orientation, self-efficacy, and innovative behavior among nurses. These findings further solidify the theoretical foundations of our study.

**TABLE 4:** Correlation analysis among core self-evaluation, error orientation, self-efficacy, and innovative behavior of nurses (*N* = 706, *r* value).

	1	2	3	4	5	6	7
1. Core self-evaluation	1						
2. Error orientation	0.469**	1					
3. Self-efficacy	0.359**	0.482**	1				
4. Idea generation	0.273**	0.422**	0.462**	1			
5. Support obtaining	0.263**	0.378**	0.510**	0.697**	1		
6. Idea realization	0.289**	0.340**	0.489**	0.598**	0.850**	1	
7. Innovative behavior	0.303**	0.417**	0.540**	0.826**	0.956**	0.915**	1

**P < 0.01.

### 3.5 Multiple linear regression analysis of factors influencing nurses’ innovative behavior

As depicted in Table 1, the one-way analysis of variance revealed significant associations between nurses’ innovative behavior and several demographic variables, including age, education level, monthly per capita household income, employment method, technical title, position, total working years (years), and whether they have published/patented/applied as the first author. Additionally, core self-evaluation, error orientation, and self-efficacy were also found to have significant correlations with innovative behavior (*P* < 0.05).

The significant variables identified from the one-way analysis of variance were subjected to multiple linear regression analysis. The results (Table 5) revealed six determinant factors associated with nurses’ innovative behavior, including position, whether to publish/patent/apply as the first author, core self-evaluation, covering up errors, thinking about errors, and self-efficacy, collectively explaining 35.9% of the variance in nurses’ innovative behavior. In addition to error cover up negatively impacting innovative behavior (β = −0.082, *P* < 0.05), all other variables positively influence innovative behavior (*P* < 0.05).

**TABLE 5:** Multiple linear regression analysis of factors influencing nurses’ innovative behavior (*N* = 706).

Variables	Partial regression coefficient	SE	Standardized partial regression coefficient	*t*	*P*	95% CI
Constant	7.349	3.025	**–**	2.429	0.015	[1.409, 13.289]
Position	0.900	0.319	0.108	2.825	0.005	[0.275, 1.526]
Whether to publish/patent/apply as the first author	1.492	0.617	0.099	2.419	0.016	[0.281, 2.704]
Core self-evaluation	0.125	0.046	0.111	2.727	0.007	[0.035, 0.214]
Covering up errors	−0.136	0.067	−0.082	−2.016	0.044	[−0.268, −0.004]
Thinking about errors	0.263	0.100	0.129	2.617	0.009	[0.066, 0.460]
Self-efficacy	0.470	0.045	0.391	10.511	<0.001	[0.382, 0.558]

*R*^2^ = 0.359, *F* = 22.970, *P* < 0.001. CI, confidence interval.

### 3.6 Testing the mediating role of error orientation and self-efficacy of nurses

Table 6 showed the results of the multiple mediating effects of error orientation and self-efficacy between core self-evaluation and innovative behavior. The total effect of core self-evaluation on innovative behavior was significant (β = 0.324, *P* < 0.001, 95% CI 0.248, 0.402). The direct effect of core self-evaluation on innovative behavior was significant (β = 0.085, *P* < 0.001, 95% CI 0.007, 0.165). The total indirect effect of core self-evaluation on innovative behavior was significant (β = 0.239, *P* < 0.001, 95% CI 0.187, 0.301), which accounted for 73.77% of the total effect. Error orientation was the mediating variable for core self-evaluation to influence innovative behavior (β = 0.097, *P* < 0.001, 95% CI 0.052, 0.161), which accounted for 29.94% of the total effect. Self-efficacy was the mediating variable for core self-evaluation to influence innovative behavior (**β** = 0.064, *P* < 0.001, 95% CI 0.023, 0.112), which accounted for 19.75% of the total effect. Error orientation and self-efficacy were the mediating variable for core self-evaluation to influence innovative behavior (β = 0.078, *P* < 0.001, 95% CI 0.053, 0.121), which accounted for 24.07% of the total effect. In summary, the results presented in Table 6 provide robust confirmation of Hypothesis 4, revealing that both error orientation and self-efficacy play significant mediating roles in the relationship between core self-evaluation and innovative behavior among nurses.

**TABLE 6:** Multiple mediating analyses of error orientation and self-efficacy between core self-evaluation and innovative behavior of nurses (*N* = 706).

	*B*	SE	*P*	95% CI
Core self-evaluation → innovation behavior (total effect)	0.324	0.039	<0.001	[0.248, 0.402]
Core self-evaluation → innovation behavior (direct effect)	0.085	0.041	0.033	[0.007, 0.165]
Core self-evaluation → innovation behavior (total indirect effect)	0.239	0.028	<0.001	[0.187, 0.301]
Core self-evaluation → error orientation	0.411	0.054	<0.001	[0.314, 0.534]
Core self-evaluation → self-efficacy	0.166	0.051	0.008	[0.059, 0.260]
Error orientation → innovation behavior	0.235	0.057	<0.001	[0.125, 0.353]
Self-efficacy → innovation behavior	0.387	0.048	<0.001	[0.284, 0.475]
Error orientation → self-efficacy	0.488	0.006	<0.001	[0.368, 0.602]
Core self-evaluation → error orientation → innovation behavior	0.097	0.016	<0.001	[0.052, 0.161]
Core self-evaluation → self-efficacy → innovation behavior	0.064	0.039	0.006	[0.023, 0.112]
Core self-evaluation → error orientation → self-efficacy → innovation behavior	0.078	0.041	<0.001	[0.053, 0.121]

### 3.7 Constructing the structural equation model

**FIGURE 1:**
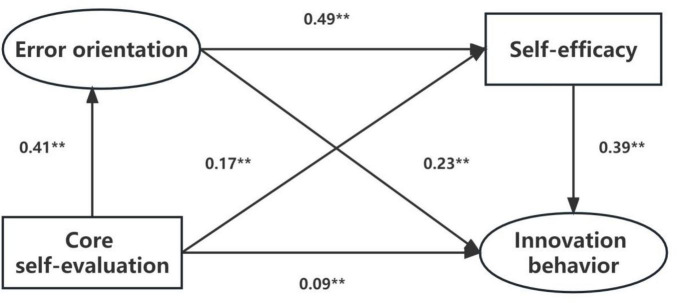
Final model. **P < 0.01.

This study established a cross layer comprehensive path analysis model based on the correlation and regression analysis of the variables. After modification, the final model was fitted to the data (χ^2^/df = 2.727, GFI = 0.993, RMSEA = 0.045, NFI = 0.988, IFI = 0.993, TLI = 0.974, CFI = 0.993, and RFI = 0.957). All the revised indexes showed that the model was well suitable and all the path coefficients reached a significant level. Core self-evaluation, error orientation and self-efficacy of nurses had a positive predictive effect on innovation behavior, with the path coefficients at 0.09, 0.23, and 0.39, respectively (*P* < 0.01) (Figure 1).

## 4 Discussion

The ultimate goal of this study is to comprehensively explore the intricate interplay between core self-evaluation, error orientation, self-efficacy, and innovative behavior among nurses. By investigating these relationships within the framework of Bandura’s social cognitive theory, our study aims to contribute valuable insights into the factors influencing nurses’ propensity for innovation. This exploration is designed to advance our understanding of the psychological determinants that shape nurses’ attitudes and behaviors, ultimately fostering a deeper comprehension of how these factors collectively impact the quality of patient care and healthcare outcomes.

### 4.1 Impact of socio-demographic on nurses’ innovative behavior

Among nurses, two statistically significant variables were identified as influencing innovative behavior. The positions held within healthcare organizations emerged as a significant factor impacting innovation opportunities and resources, particularly for those in leadership roles, consistent with Ayvaz’s findings (Yıldız Ayvaz et al., 2019). Active engagement in academic or research activities, seeking publication or patents, reflects a proactive and innovative mindset. This stems from desires for professional growth, institutional encouragement, and personal fulfillment as first authors. Such proactive engagement advances the nursing profession. Recognizing and nurturing these factors, healthcare organizations can cultivate an environment promoting innovation for improved patient care and outcomes. Moreover, core self-evaluation, error orientation, and self-efficacy impact nurses’ innovation. The participants’ medium-high levels in these areas suggest a positive, supportive environment encouraging creativity and novel problem-solving approaches in professional practice.

### 4.2 Effect of the core self-evaluation on innovative behavior

Our study unveiled a significant positive correlation between core self-evaluation and nurses’ innovative behavior, echoing Attiq and Saeed’s findings that emphasized core self-evaluation’s role in promoting innovation (Attiq et al., 2017; Saeed et al., 2019). Nurses with a positive core self-evaluation tend to possess heightened self-confidence and belief in their abilities, nurturing an innovative mindset. In nursing, this positivity empowers nurses to challenge norms, seek new approaches, and adopt inventive solutions for patient care and healthcare processes (Sulaiman et al., 2021). A robust sense of self-efficacy propels nurses to tackle challenges, experiment with ideas, and adapt to changes, fostering an innovative nursing culture. Educators and leaders shape core self-evaluation, influencing innovative behavior. Their role modeling can inspire creative thinking and idea generation (Farèiæ et al., 2020). Creating supportive learning environments that cultivate self-confidence and self-efficacy empowers nurses, driving innovation and continuous improvement. Positive core self-evaluation encourages proactive and innovative mindsets, leading to enhanced patient care and nursing progress. In conclusion, cultivating positive core self-evaluation among nurses through education and leadership interventions can foster innovation and excellence in nursing practice.

### 4.3 Effect of error orientation on innovative behavior

Our study demonstrated that error orientation significantly impacts nurses’ innovative behavior. Within our study, two dimensions of error orientation, namely “covering up errors” and “thinking about errors,” were found to influence nurses’ innovative behavior. Specifically, “covering up errors” negatively affects nurses’ innovative behavior. This dimension reflects nurses’ tendency to hide or conceal their errors (Calabrese and Selby, 2022). A higher inclination toward error cover-up might hinder open acknowledgment of mistakes, obstructing opportunities for improvement and innovation. This behavior could arise from fears of repercussions related to error disclosure. Establishing a culture of psychological safety within healthcare organizations becomes essential to address this. Fostering an environment that encourages transparent error reporting empowers nurses to share experiences, learn from errors, and drive innovation. On the other hand, “thinking about errors,” characterized by a reflective and analytical approach toward errors, positively influences nurses’ innovative behavior. Nurses adopting error thinking view mistakes as chances for learning and growth, rather than sources of blame or fear. This mindset encourages continuous improvement and prompts nurses to explore novel solutions to healthcare challenges. Existing research supports the idea that thinking about errors is linked to increased nurse innovation. For instance, Belyaeva et al. (2021) found that nurses with strong error thinking engaged in creative problem-solving and proposed innovative solutions. Similarly, Lee et al. (2017) noted a positive correlation between error thinking and nurses’ willingness to experiment with new ideas for improving patient care. In conclusion, our study underscores error orientation’s importance in shaping nurses’ innovative behavior. It highlights the need to address tendencies toward error cover-up and encourage error thinking among nurses. Cultivating a culture that learns from mistakes is crucial for promoting innovation.

### 4.4 Effect of self-efficacy on innovative behavior

Our study’s findings emphasize self-efficacy’s positive influence on nurses’ innovative behavior, aligning with Jønsson et al.’s (2021) research. Nurses with high self-efficacy exhibit confidence, viewing challenges as growth opportunities and actively seeking innovative solutions. This is consistent with Javed et al. (2021), who found self-efficacy mediating between inclusive leadership and innovation, indicating leaders’ role in fostering self-efficacy and innovation. Our study expands on Park’s (2018) work, demonstrating self-efficacy’s importance across professions. High self-efficacy relates to greater innovative behavior, critical in driving innovation, including nursing. Our study further highlights self-efficacy’s mediating role in nursing innovation, observed in other fields too (Xiang et al., 2023). This underscores its universal significance in promoting innovation. Interventions to enhance nurses’ self-efficacy could improve innovative behavior, benefiting patient care. In summary, self-efficacy significantly impacts nurses’ innovative behavior, suggesting interventions to bolster self-efficacy for enhanced patient care and innovative practices.

### 4.5 Exploring the nexus: core self-evaluation, error orientation, self-efficacy, and nurses’ innovative behavior in healthcare settings

This study investigated how error orientation and self-efficacy mediate the relationship between core self-evaluation and nurses’ innovative behavior. The results strongly endorse our hypotheses and offer valuable insights into the pivotal factors that shape nurses’ proclivity for innovation. Moreover, our study unravels the mediating roles of error orientation and self-efficacy in this dynamic relationship. Error orientation emerged as a key mediator between core self-evaluation and innovative behavior. Nurses who embrace errors as opportunities for learning demonstrated a higher degree of innovativeness, shedding light on the critical significance of fostering positive attitudes toward errors in the pursuit of innovation (Zhou et al., 2023). Similarly, self-efficacy emerged as another influential mediator. Nurses with elevated self-efficacy levels exhibited a heightened propensity for innovation, underscoring the paramount importance of bolstering their self-confidence (Li et al., 2022). Notably, the combination of a positive error orientation and high self-efficacy further amplified the impact of core self-evaluation on innovation. This emphasizes the importance of promoting positive self-perception, confidence, and constructive error attitudes to nurture innovation. Healthcare institutions can then create a supportive environment that unleashes the innovative potential of their nursing staff.

## 5 Implications for nursing

The findings of this study hold important implications for nursing practice and leadership. Nurse administrators and managers should prioritize strategies that enhance nurses’ self-efficacy beliefs. By fostering nurses’ confidence in their abilities and supporting their decision-making, healthcare organizations can empower nurses to take on innovative approaches in patient care and problem-solving. Furthermore, creating a culture that values and encourages innovation is essential. Nurse leaders should actively promote a positive error orientation, where mistakes are viewed as learning opportunities rather than sources of blame. By nurturing a learning-oriented environment, nurses are more likely to be open to exploring new ideas and engaging in innovative practices. In conclusion, the study emphasizes the significance of self-efficacy and a positive error orientation in fostering innovative behavior among nurses. Nurse leaders can play a pivotal role in cultivating a supportive and innovative culture that empowers nurses to contribute meaningful innovations to patient care and healthcare practices.

## 6 Limitations

This study has limitations that warrant consideration. The cross-sectional design and convenience sampling employed may restrict the generalizability of findings, lacking temporal insights and potentially limiting representation. Future research could benefit from longitudinal designs and diverse sampling methods for enhanced applicability. The use of online questionnaires introduces potential biases related to respondent preferences and internet access, suggesting the potential for mixed-methods approaches to offer a more comprehensive understanding. Additionally, the lack of control for variables in mediation analysis may impact reliability, emphasizing the need for future studies to incorporate a more comprehensive set of variables for a nuanced understanding. In summary, future research should explore longitudinal and diverse sampling approaches, mixed-methods designs, and more robust mediation analyses to address these limitations and deepen our understanding of the phenomena under investigation.

## 7 Conclusion

The study reveals intricate connections among core self-evaluation, error orientation, self-efficacy, and innovative behavior in the nursing context. Positive core self-evaluation is crucial for nurturing innovative practices among nurses, playing a dual role by directly influencing innovative behavior and mediating the relationship between error orientation and self-efficacy. Cultivating a positive core self-evaluation acts as a foundation for fostering innovative behaviors, empowering nurses to navigate challenges, learn from mistakes, and exhibit a higher propensity for innovation. Our conclusions emphasize the need for a holistic approach to enhance nurses’ innovative behavior, suggesting that managers and administrators can benefit by implementing strategies that promote positive core self-evaluation while addressing the broader aspects of error orientation and self-efficacy. This comprehensive perspective aligns with the central theme of our research, highlighting the importance of prioritizing and fostering innovative behaviors among nurses for a more resilient and dynamic healthcare environment.

## Data availability statement

The raw data supporting the conclusions of this article will be made available by the authors, without undue reservation.

## Ethics statement

The studies involving humans were approved by the Ethics Committee of Zhejiang University of Traditional Chinese Medicine. The studies were conducted in accordance with the local legislation and institutional requirements. The participants provided their written informed consent to participate in this study.

## Author contributions

GM: Writing – original draft and Investigation. ZH: Writing – review & editing. XM: Writing – review & editing.

## References

[B1] Asurakkody T. A. Shin S. Y. (2018). Innovative behavior in nursing context: A concept analysis. *Asian Nurs. Res.* 12 237–244. 10.1016/j.anr.2018.11.003 30471386

[B2] Attiq S. Wahid S. Javaid N. Kanwal M. Shah H. J. (2017). The impact of employees’ core self-evaluation personality trait, management support, co-worker support on job satisfaction, and innovative work behaviour. *Pak. J. Psychol. Res.* 32:247.

[B3] Yıldız Ayvaz M. Akyol Y. E. Demiral M. (2019). Innovation in nursing and innovative attitudes of nurses. *Int. J. Health Administr. Educ. Congr.* 5 52–59.

[B4] Bandura A. Freeman W. H. Lightsey R. (1999). Self-efficacy: The exercise of control. *J. Cogn. Psychother.* 13 156–166. 10.1891/0889-8391.13.2.158 11261958

[B5] Belyaeva O. V. Vysotskaya N. V. Gazizova A. I. Kurbatskaya T. B. Myavlina N. J. (2021). “The methodology of design thinking as a tool for forming innovative solutions,” in *Frontier information technology and systems research in cooperative economics. Studies in systems, decision and control*, Vol. 316 eds Bogoviz A. V. Suglobov A. E. Maloletko A. N. Kaurova O. V. Lobova S. V. (Cham: Springer). 10.1007/978-3-030-57831-2_26

[B6] Cross B. J. Collard J. J. Levidi M. D. C. (2021). Core self-evaluation, rumination and forgiveness as an influence on emotional distress. *Curr. Psychol.* 42 2087–2099. 10.1007/s12144-021-01628-4

[B7] Calabrese E. J. Selby P. B. (2022). Cover up and cancer risk assessment: Prominent US scientists suppressed evidence to promote adoption of LNT. *Environ. Res.* 210:112973. 10.1016/j.envres.2022.112973 35182593

[B8] Chen H. Liu F. Wen Y. (2022). The influence of college students’ core self-evaluation on job search outcomes: Chain mediating effect of career exploration and career adaptability. *Curr. Psychol.* 10.1007/s12144-022-02923-4 [Epub ahead of print]. 35228786 PMC8865730

[B9] Drury A. Sulosaari V. Sharp L. Ullgren H. de Munter J. Oldenmenger W. (2023). The future of cancer nursing in Europe: Addressing professional issues in education, research, policy and practice. *Eur. J. Oncol. Nurs.* 63:102271. 10.1016/j.ejon.2023.102271 36827835

[B10] Farèiæ N. Baraæ I. Plužariæ J. Ilakovac V. Paèariæ S. Gvozdanoviæ Z. (2020). Personality traits of core self-evaluation as predictors on clinical decision-making in nursing profession. *PLoS One* 15:e233435. 10.1371/journal.pone.0233435 32421752 PMC7233533

[B11] Farnese M. L. Fida R. Picoco M. (2022). Error orientation at work: Dimensionality and relationships with errors and organizational cultural factors. *Curr. Psychol.* 41 970–989. 10.1007/s12144-020-00639-x

[B12] Javed B. Fatima T. Khan A. K. Bashir S. (2021). Impact of inclusive leadership on innovative work behavior: The role of creative self-efficacy. *J. Creat. Behav.* 55 769–782. 10.1002/jocb.487

[B13] Jønsson T. F. Bahat E. Barattucci M. (2021). How are empowering leadership, self-efficacy and innovative behavior related to nurses’ agency in distributed leadership in Denmark, Italy and Israel. *J. Nurs. Manag.* 29 1517–1524. 10.1111/jonm.13298 33641199

[B14] Judge T. A. Bono J. E. (2001). Relationship of core self-evaluations traits-self-esteem, generalized self-efficacy, locus of control, and emotional stability-with job satisfaction and job performance: A meta-analysis. *J. Appl. Psychol.* 86 80–92. 10.1037/0021-9010.86.1.80 11302235

[B15] Lee D. S. Abdullah K. L. Subramanian P. Bachmann R. T. Ong S. L. (2017). An integrated review of the correlation between critical thinking ability and clinical decision-making in nursing. *J. Clin. Nurs.* 26 4065–4079. 10.1111/jocn.13901 28557238

[B16] Li X. Cheng M. Xu J. (2022). Leaders’ innovation expectation and nurses’ innovation behaviour in conjunction with artificial intelligence: The chain mediation of job control and creative self-efficacy. *J. Nurs. Manag.* 30 3806–3816. 10.1111/jonm.13749 35899457

[B17] Ling B. Lin W. Ya-qing Z. (2012). Development and analysis of reliability and validity of nurse innovative behavior scale. *J. Shanghai Jiaotong Univ.* 32 1079–1082. 10.3969/j.issn.1674-8115.2012.08.025

[B18] Ng T. W. Lucianetti L. (2016). Within-individual increases in innovative behavior and creative, persuasion, and change self-efficacy over time: A social–cognitive theory perspective. *J. Appl. Psychol.* 101 14–34. 10.1037/apl0000029 26052714

[B19] Park H. (2018). The effect of nurse’s coaching leadership on self-efficacy, job engagement and innovative behavior in hospital. *J. Korea Contents Assoc.* 18 260–272. 10.5392/JKCA.2018.18.09.260

[B20] Pennington G. Driscoll A. (2019). Improving retention of home health nurses: Fostering outcome sustainability through an innovative orientation and mentorship program. *Home Healthc. Now* 37 256–264. 10.1097/NHH.0000000000000782 31483357

[B21] Rybowiak V. Garst H. Frese M. Batinic B. (1999). Error orientation questionnaire (EOQ): Reliability, validity, and different language equivalence. *J. Organ. Behav.* 20 527–547.

[B22] Saeed B. B. Afsar B. Cheema S. Javed F. (2019). Leader-member exchange and innovative work behavior: The role of creative process engagement, core self-evaluation, and domain knowledge. *Eur. J. Innov. Manage.* 22 105–124. 10.1108/EJIM-11-2017-0158

[B23] Schwarzer R. (1997). Optimistic self-beliefs: Assessment of general perceived self-efficacy in thirteen cultures. *World Psychol.* 3 177–190.

[B24] Sulaiman A. M. Alfuqaha O. A. Shaath T. A. Alkurdi R. I. Almomani R. B. (2021). Relationships between core self-evaluation, leader empowering behavior, and job security among Jordan University Hospital nurses. *PLos One* 16:e260064. 10.1371/JOURNAL.PONE.0260064 34788327 PMC8598013

[B25] Weiwei G. (2008). *The relationship between entrepreneurial error orientation and entrepreneurial performance based on action theory.* Zhejiang: Zhejiang University.

[B26] Xiang D. Ge S. Zhang Z. Budu J. T. Mei Y. (2023). Relationship among clinical practice environment, creative self-efficacy, achievement motivation, and innovative behavior in nursing students: A cross-sectional study. *Nurse Educ. Today* 120:105656. 10.1016/j.nedt.2022.105656 36436269

[B27] Xu H. Jin H. Li H. (2022). The relationship between leaders’ long-term orientation and employees’ innovative behaviors. *Creat. Res. J.* 10.1080/10400419.2022.2124357 [Epub ahead of print].

[B28] Yun C. Du P. (2019). The influence of core self-evaluation and error orientation on employees’ innovative behavior. *J. Capital Univ. Econ. Bus.* 21 100–108. 10.13504/j.cnki.issn1008-2700.2019.06.010

[B29] Zhang Z. J. (2005). Handbook of scales for behavioral medicine. *Chin. Med. Multimedia Press* 2005 332–335.

[B30] Zhou X. Wang Y. Zhang X. Li L. (2023). The influence of decision-making logic on employees’ innovative behaviour: The mediating role of positive error orientation and the moderating role of environmental dynamics. *Psychol. Res. Behav. Manag.* 16 2297–2313. 10.2147/PRBM.S416595 37383418 PMC10296618

